# Bactericidal Activity of Curcumin I Is Associated with Damaging of Bacterial Membrane

**DOI:** 10.1371/journal.pone.0121313

**Published:** 2015-03-26

**Authors:** Poonam Tyagi, Madhuri Singh, Himani Kumari, Anita Kumari, Kasturi Mukhopadhyay

**Affiliations:** School of Environmental Sciences, Jawaharlal Nehru University, New Delhi, 110067, India; Beijing Institute of Microbiology and Epidemiology, CHINA

## Abstract

Curcumin, an important constituent of turmeric, is known for various biological activities, primarily due to its antioxidant mechanism. The present study focused on the antibacterial activity of curcumin I, a significant component of commercial curcumin, against four genera of bacteria, including those that are Gram-positive (*Staphylococcus aureus* and *Enterococcus faecalis)* and Gram-negative (*Escherichia coli* and *Pseudomonas aeruginosa)*. These represent prominent human pathogens, particularly in hospital settings. Our study shows the strong antibacterial potential of curcumin I against all the tested bacteria from Gram-positive as well as Gram-negative groups. The integrity of the bacterial membrane was checked using two differential permeabilization indicating fluorescent probes, namely, propidium iodide and calcein. Both the membrane permeabilization assays confirmed membrane leakage in Gram-negative and Gram-positive bacteria on exposure to curcumin I. In addition, scanning electron microscopy and fluorescence microscopy were employed to confirm the membrane damages in bacterial cells on exposure to curcumin I. The present study confirms the broad-spectrum antibacterial nature of curcumin I, and its membrane damaging property. Findings from this study could provide impetus for further research on curcumin I regarding its antibiotic potential against rapidly emerging bacterial pathogens.

## Introduction

Curcumin is a natural component of the rhizome *Curcuma longa*, also known as turmeric. Since ancient times, the root of turmeric has been popular as a spice in India and other Asian countries. It is also commonly used for medicinal purposes, particularly to treat inflammatory conditions [1]. Over the years, curcumin has been explored for various clinical applications. Previous research has shown that it plays a therapeutic role in many diseases including diabetes, inflammatory disorders and different types of cancers [2–4]. Curcumin is a pleiotropic molecule that interacts with multiple targets involved in inflammatory reaction such as tumor necrosis factor-alpha (TNFα) and interleukins (ILs) [5]. Curcumin has also been shown to possess *in vitro* anti-microbial potential against a wide range of microorganisms including fungi [6] as well as several Gram-positive and Gram-negative bacteria [7–17]. Recently, Song *et al*. [9] showed that curcumin suppresses adherence of *Streptococcus* mutants to human tooth surfaces and extra-cellular matrix protein. Research has further highlighted that curcumin possesses a synergistic effect with important antibiotics such as cefixime, vancomycin and tetracycline against *Staphylococcus aureus* (*S*. *aureus*) [10–12]. However, very few studies have demonstrated the mechanism of antibacterial activity of curcumin I which seems to differ depending on the strain being studied. For instance, studies have demonstrated that the antibacterial activity of curcumin against *Bacillus subtilis* occurs through the inhibition of bacterial cell proliferation by blocking the assembly dynamics of FtsZ in the Z ring [15, 16]. In the case of *Pseudomonas aeruginosa* (*P*. *aeruginosa*) infection, curcumin was shown to have anti-infective activity through affecting virulence, quorum sensing and biofilm initiation [17]. Moreover, these mechanisms have not been confirmed in the case of other bacterial genera, hence could not be generalized for all bacteria. Therefore, a detailed study on antibacterial mechanism of curcumin, including a large number of bacteria from different genera is required. Due to the increase of resistance in Gram-positive and Gram-negative bacteria, there is an urgent need to identify and assess alternative antimicrobials, including those from plant materials with low human cytotoxicity. Curcumin I showed no toxic effect on human health even when taken at doses as high as 8 g day^−1^ [18, 2]. Considering these factors, in the present study, we investigated the detailed antibacterial activity of curcumin I (the major component of commercial curcumin) against two Gram-positive bacteria, namely, *S*. *aureus* and *Enterococcus faecalis* (*E*. *faecalis*), and two Gram-negative bacteria, namely, *Escherichia coli* (*E*. *coli*) and *P*. *aeruginosa*. We extended our study to explore the bacterial membrane lysis induced by curcumin I against the organisms from the mentioned bacterial groups. The membrane lysing properties of curcumin were further viewed through scanning electron microscopy and confocal fluorescence microscopy.

## Materials and Methods

### Materials

Curcumin I (purity ≥ 97%) and brain heart infusion (BHI) media were purchased from Himedia, India. Human neutrophil peptide-1 (HNP-1), nisin, gramicidin D, calcein-AM (solubilized in dimethyl sulfoxide (DMSO)) and propidium iodide (PI) were procured from Sigma-Aldrich, USA. Glutaraldehyde was ordered from Merck, Germany. The stock concentration of curcumin I was made in DMSO, and the working concentrations were diluted in PBS for killing assays in such a way that the DMSO concentration remains <1%.

### Microbial strains

Two Gram-positive bacteria; *S*. *aureus* ATCC 29213 and *E*. *faecalis* ATCC 29212, and two Gram-negative bacteria; *E*. *coli* ATCC 25922 and *P*. *aeruginosa* ATCC 25619, were used in this study. These strains were stored at −80°C in 15% (v/v) glycerol until sub cultured onto BHI agar plate for further studies.

### Killing assay

The killing assay was performed according to the procedure mentioned in our previous publications [19, 20]. Bacterial cells were grown in BHI broth to mid logarithmic phase. Using spectrophotometer, the optical density (OD 600_nm_) of cells was adjusted to 0.5 (~10^8^ CFU/ml) in 10 mM PBS buffer (pH 7.4). A final inoculum of 10^6^ and 10^4^ CFU/ml was exposed to various concentrations of curcumin I (25 μM, 50 μM & 100 μM) in the presence of PBS [19]. At the chosen time points (30 min, 60 min & 120 min), aliquots were taken, placed on BHI agar plate in triplicates and incubated overnight at 37°C. Next day, the bacterial colonies were counted and bacterial survival was expressed as the mean percentage of survival versus non-peptide treated control (set as 100% survival). Each experiment was repeated independently on three separate days.

### Membrane permeabilization assay

Permeabilization of the membrane of *S*. *aureus* and *E*. *coli* following exposure to curcumin I was determined, using both steady-state fluorescence and flow cytometry techniques. Two strategic assays were performed: (a) uptake of extracellular PI by permeabilized cells, and (b) leakage of preloaded calcein from the permeabilized cells.

### Propidium iodide (PI) uptake assay

PI intercalates with bases of deoxyribonucleic acid (DNA) and gives fluorescence. PI can enter the bacterial cell membrane only when it has been permeabilized through an agent, and bind with DNA. This DNA-bound PI fluoresces with excitation and emission at 544 nm and 620 nm, respectively. The fluorescence emission can be detected by both spectrofluorimeter [19] and flow cytometer [21] equipped with an appropriate filter. Briefly, *S*. *aureus* and *E*. *coli* cells were grown in BHI broth up to the mid logarithmic phase, harvested, washed, and adjusted to 10^6^ CFU/ml in PBS buffer. The cells were incubated at 37°C with curcumin I (25 μM, 50 μM & 100 μM) and simultaneously with two bacterial membrane permeabilizing agents HNP-1 and nisin (both have been used as positive controls) at indicated time points. After curcumin exposure, the cells were washed in PBS buffer and incubated with PI (1.3 μg/ml) at 37°C for 20 min in dark. The PI fluorescence was measured at excitation and emission of 544 nm and 620 nm respectively, through a fluorescence spectrophotometer (Shimadzu RF-5301 PC spectrofluorimeter, Japan). Similarly, separate aliquots were prepared and measured in the flow cytometer (Becton Dickinson (BD) Facs verse, San Jose, CA, and Beckman Coulter, CA). A total of 10,000 cells were acquired for each flow cytometry analysis.

### Calcein leakage assay

The permeabilization of bacterial membrane due to curcumin I exposure was measured via calcein leakage by flow cytometry, as described in our previous reports [19, 20]. Calcein-AM (Calcein-Acetoxy Methyl Ester) is a membrane-permeating, non-fluorescent derivative of calcein whose excitation and emission wavelengths are 490 nm and 517 nm, respectively. After entering into the cell, calcein-AM is cleaved by cytoplasmic esterases, releasing the fluorescent calcein, which is membrane-impermeable. Calcein can leak out of the cells only if their membrane is damaged. In brief, mid log *S*. *aureus* cells were loaded with calcein by incubating 1 ml of cells (10^8^ CFU/ml) with 2 μg/ml of calcein-AM at 37°C for 2h. The *S*. *aureus* cells loaded with calcein were diluted 100-fold (10^6^ CFU/ml), treated with different concentrations of curcumin I (i.e., 25 μM, 50 μM & 100 μM) and gramicidin D, a well known pore forming antimicrobial peptide [20] for 2h at 37°C. Untreated calcein loaded cells were taken as negative control (no calcein leakage) and 13 μM gramicidin D treated cells were kept as positive control. Flow cytometry was performed and a total of 10,000 cells were acquired for each flow cytometry analysis. *S*. *aureus* cells at or above the threshold of 10^2^ fluorescence units (FL units) were considered to have retained calcein, indicative of an intact cytoplasmic membrane. Those cells showing <10^2^ FL units were considered to have lost calcein due to curcumin I exposure. Differences in the levels of membrane permeabilization were defined as the percentages of difference in cell fluorescence units between curcumin I treated and untreated (control) samples. The release of calcein from curcumin I and gramicidin D treated samples was measured on three different days.

### Scanning electron microscopy (SEM)

SEM was performed as described in our previous report [20]. In brief, 10^8^ CFU/ml bacterial suspension was incubated with different doses of curcumin I (25 μM, 50 μM & 100 μM) in PBS for 60 min and centrifuged at 4,000 rpm for 10 min. It was washed with PBS (pH 7.4) two times and fixed overnight at 4°C with 2.5% glutaraldehyde in 0.1 M PBS (pH 7.4). Cells were washed two–three times in double distilled water and dehydrated in series of graded ethanol (20%–90%). The dehydrated cells were finally dissolved in 100% ethanol and dried under vacuum. The cells were coated with 15 nm gold particles through automatic sputter coater. The samples were then viewed through SEM (Zeiss EVO 40).

### Microscopic evaluation of bacterial (membrane damage) viability

Bacterial viability and membrane integrity were analyzed using the LIVE/DEAD *Bac*Light Bacterial Viability Assay Kit for microscopy [L7007, from Invitrogen, this is a mixture of stains: SYTO 9 (for live cells) and PI (for dead cells) in two different ratios] according to the protocol as suggested by the manufacturer [19]. Briefly, 3 μl of the mixture was added to the bacterial cells previously treated with different concentrations of curcumin I (25 μM, 50 μM & 100 μM) for 2h, and untreated cells were kept as control. The samples were incubated for 15 min in dark at room temperature and 5 μl of this sample was trapped in between coverslip and glass slide. The slide was viewed under a confocal fluorescence microscope (Olympus Fluoview FV 1000 model), using 60X objective sequentially using fluorescence setting for FITC (green/ syto 9 +ve cells) and PI (red/PI +ve cells) filters, respectively, followed by phase contrast and bright field settings. Images were acquired and analyzed using Olympus Fluoview version 2.1 software for quantification of live (green fluorescence) and dead (red fluorescence) cells.

### Statistical analysis

All the killing experiments were performed in triplicate, repeated in three independent experiments on different days and plotted as mean ± SD. The rest of the assays were performed as three independent experiments on different days and plotted as mean ± SD. Statistical analysis (multiple comparison among data sets) was performed using one-way analysis of variance (ANOVA) using Minitab [19, 20]. A p value ≤ 0.05 was considered significant.

## Results

### Killing activity of curcumin I against bacteria from different genera

**Fig 1:**
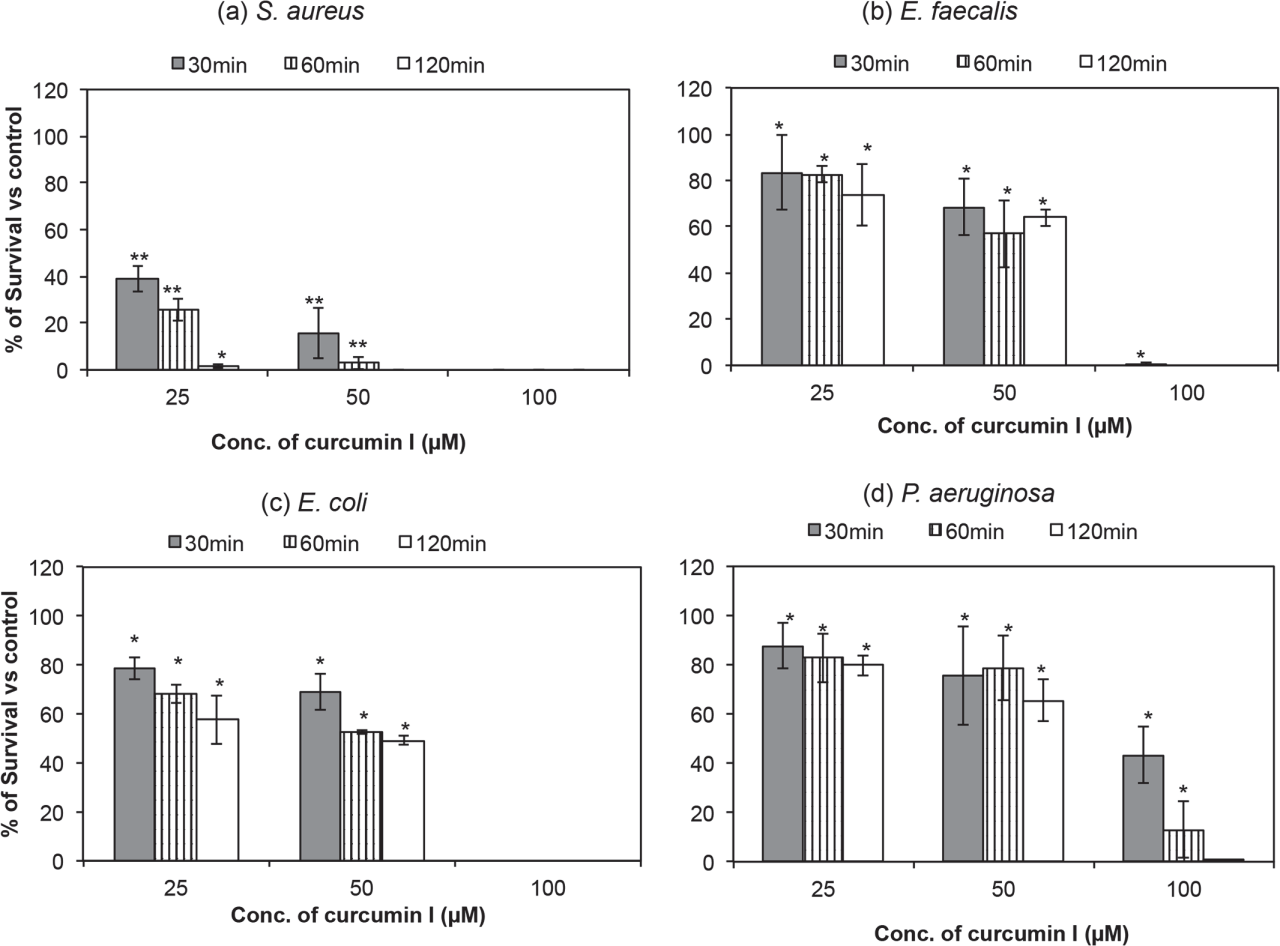
Time kill kinetics of curcumin I against 10^4^ CFU/ml of four different bacterial cells. Killing bars for (a) *S*. *aureus* ATCC 29213, (b) *E*. *faecalis* ATCC 29212, (c) *E*. *coli* ATCC 25922, and (d) *P*. *aeruginosa* ATCC 25619 by 25 μM, 50 μM & 100 μM curcumin I after 30, 60 & 120 min of incubation. Symbols: Gray, vertical striped and white represent 30, 60 & 120 min exposure of curcumin I, respectively. These data represent mean (±SD) of three independent experiments (*p ≤ 0.001, **p ≤ 0.01, ***p ≤ 0.05).

The antibacterial activity of curcumin I against two Gram-positive bacteria (*S*. *aureus* & *E*. *faecalis*) and two Gram-negative bacteria (*E*. *coli* & *P*. *aeruginosa*) was examined by killing assays performed after exposing the cells (10^4^ CFU/ml) to three different concentrations of curcumin I (25 μM, 50 μM & 100 μM) for 2h and plating aliquots at selected time points (30 min, 60 min & 120 min) on BHI agar plate. The percentage of survival of curcumin I treated bacterial samples was calculated after comparing them with control (untreated) and is presented in Fig. 1A–1D. Curcumin I showed a strong killing potential against all the tested microorganisms, namely, *S*. *aureus* (Fig. 1A), *E*. *faecalis* (Fig. 1B), *E*. *coli* (Fig. 1C) and *P*. *aeruginosa* (Fig. 1D). The killing increased with an increase in the dosage of curcumin I as well as exposure time. For instance, exposure to 25 μM curcumin I for 30 min showed 12%–60% killing of all the tested bacteria, while the same concentration of curcumin I showed 20%–92% killing after 2h of incubation against all the four genera. Similarly, a 30 min exposure to 100 μM of curcumin I showed a 100 ± 0.0%, 99 ± 0.6%, 100 ± 0.0% and 57 ± 11.5% killing against *S*. *aureus*, *E*. *faecalis*, *E*. *coli* and *P*. *aeruginosa*, respectively. The killing reached 100% after 2h long exposure to 100 μM curcumin I. The variation in curcumin I’s killing efficacy at different concentrations and exposure durations was found to be statistically significant (p value < 0.05 on comparing percentage survival versus concentration).

### Killing of *S*. *aureus* and *E*. *coli* by curcumin I at higher cell density

**Fig 2:**
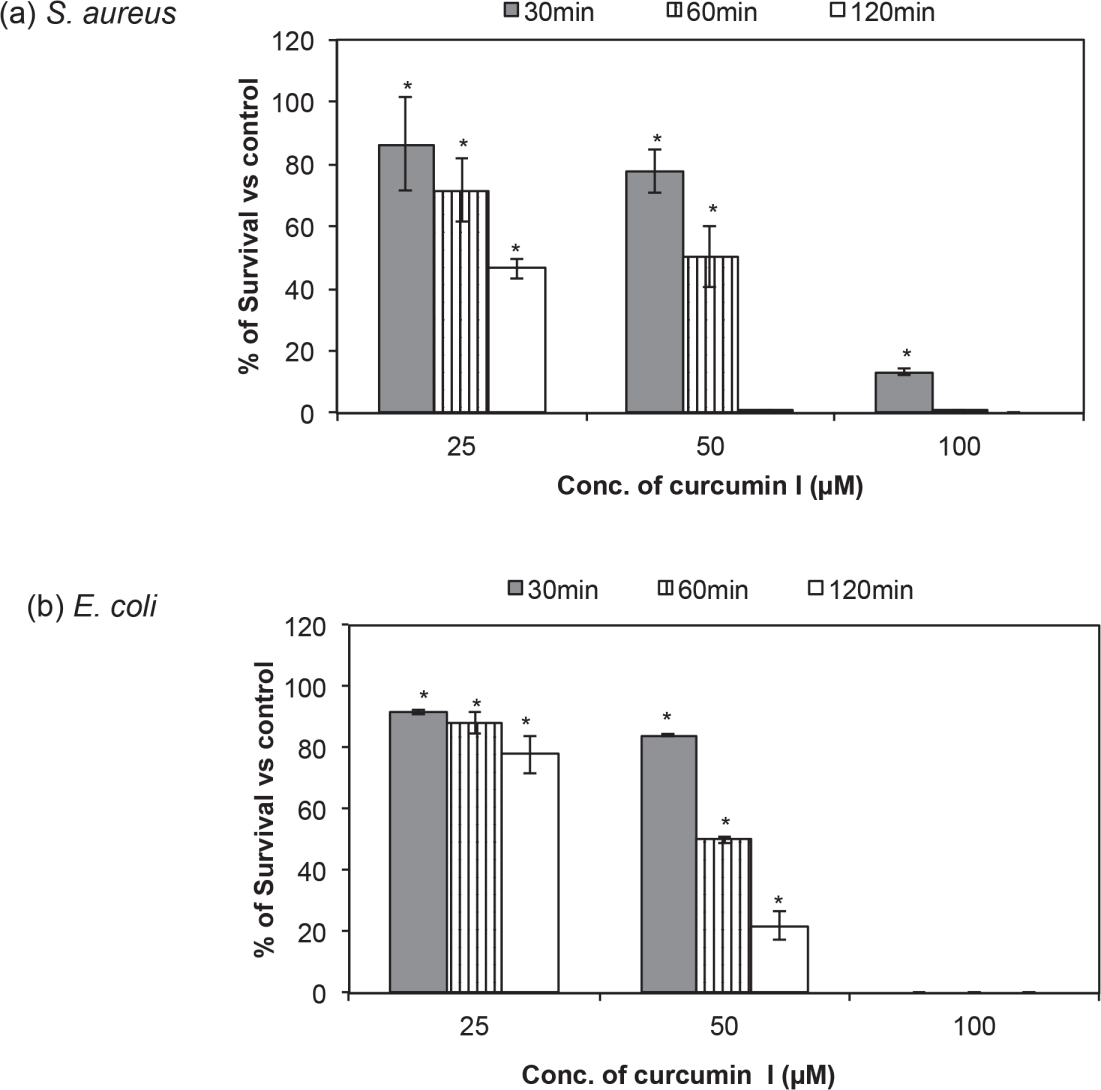
Time kill kinetics of curcumin I against 10^6^ CFU/ml of *S*. *aureus* and *E*. *coli* cells. Killing bars for (a) *S*. *aureus* ATCC 29213, and (b) *E*. *coli* ATCC 25922 by different concentrations of curcumin I (25 μM, 50 μM & 100 μM) after 30, 60 & 120 min of incubation. Gray, vertical striped and white represent 30, 60 & 120 min exposure of curcumin I, respectively. These data represent mean (±SD) of three independent experiments (*p ≤ 0.001).

This study determined the effect of higher inoculums (10^6^ CFU/ml) of *S*. *aureus* and *E*. *coli* on the killing activity of curcumin I at concentrations of 25 μM, 50 μM and 100 μM. The percentages of survival of *S*. *aureus* and *E*. *coli* cells are presented in Fig. 2A and 2B, respectively. The results show that the efficacy of curcumin I did not reduce when a higher (10^6^ CFU/ml) inoculum of *S*. *aureus* (Fig. 2A) cells was used. For example, 25 μM of curcumin I showed 14 ± 14.8%, 28 ± 10.1% and 53 ± 3.3% killing against *S*. *aureus* at the time points of 30 min, 60 min and 120 min, respectively. Further increases in the concentration of curcumin I increased the killing efficacy. Thus, exposure to 100 μM of curcumin I resulted in 87 ± 1.1%, 99 ± 0.4% and 100 ± 0.0% killing activity at the time points of 30 min, 60 min and 120 min, respectively, against *S*. *aureus*. Similar patterns of killing were also noticed in cases of higher inoculum of *E*. *coli* (Fig. 2B). For example, exposure to 25 μM of curcumin I showed 8 ± 0.9%, 12 ± 3.4% and 22 ± 6.3% killing at the time points of 30 min, 60 min and 120 min, respectively, and exposure to 100 μM of curcumin I resulted in 100 ± 0.0% killing at all the time points.

### Membrane permeabilization of *S*. *aureus* by PI uptake assay

**Fig 3:**
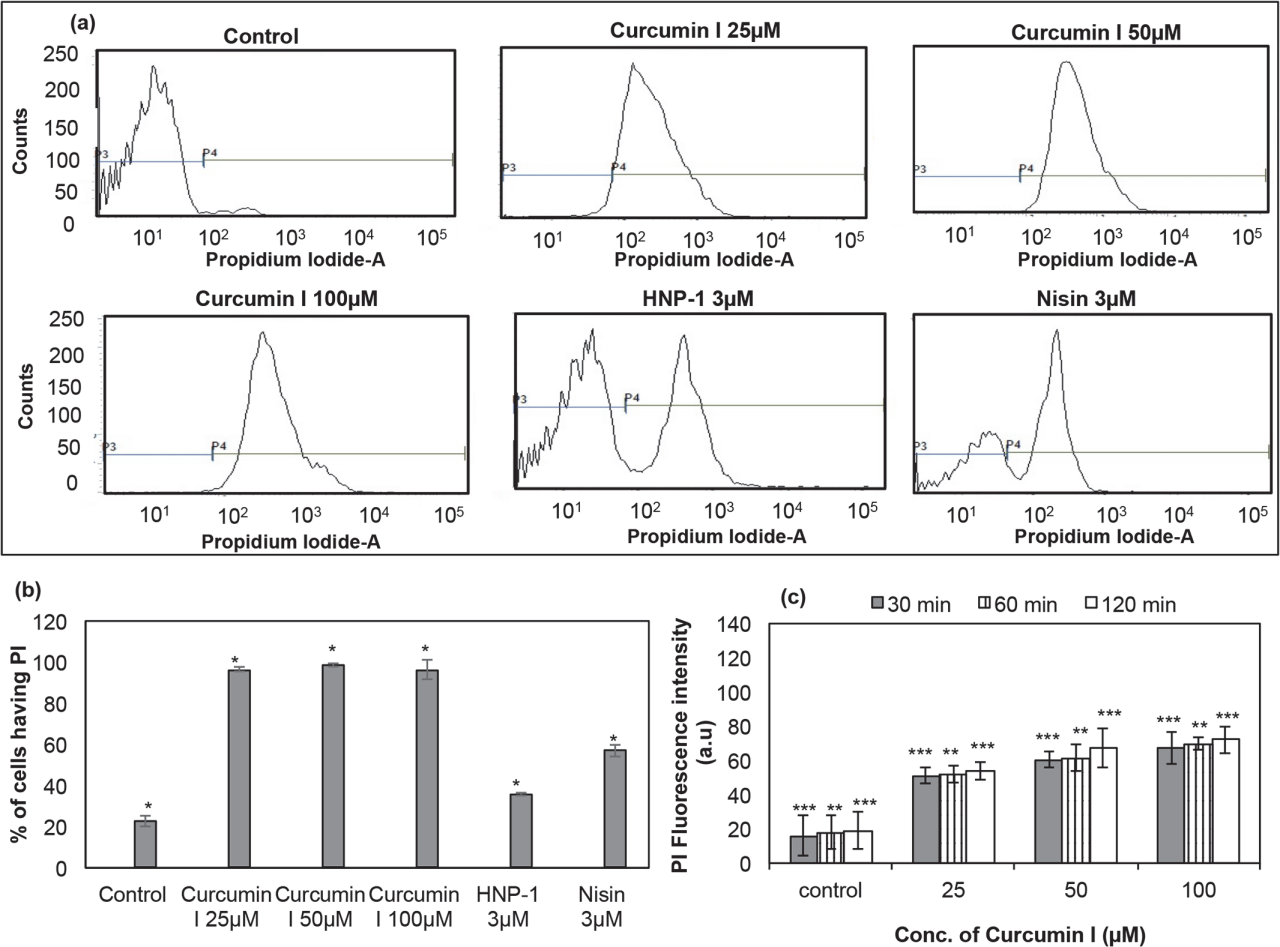
Membrane permeabilization of *S*. *aureus* by curcumin I, using propidium iodide (PI) through flow cytometry and spectrofluorimetry technique. A total of 10,000 cells were acquired for each flow cytometry analysis. (a) Histograms of logarithmic *S*. *aureus* ATCC 29213 cells labeled with PI and treated with different concentrations of curcumin I (0 μM (control), 25 μM, 50 μM & 100 μM), and positive controls (HNP-1 and nisin) for 2h, (b) percentage of PI uptake by *S*. *aureus* as quantified from flow cytometry data and (c) fluorescence intensity of PI in *S*. *aureus* cells exposed to curcumin I using spectrofluorimetry. These data represent mean (±SD) of three independent experiments (*** p ≤ 0.05, ** p ≤ 0.01,* p < 0.001).

To delineate the mechanism behind the antibacterial activity of curcumin I, its ability to permeabilize the bacterial membrane was examined by PI uptake assay. For this purpose, initially *S*. *aureus* ATCC 29213 was chosen (Fig. 3A–3C). As shown in Fig. 3A, untreated cells did not display any PI fluorescence intensity (Fluorescence unit < 10^2^ arbitrary units (a.u.) was set as threshold fluorescence). On the other hand, *S*. *aureus* cells treated with curcumin I showed PI fluorescence intensity > 10^2^ a.u. This signature was prominent in case of higher concentration of curcumin I. The increase in fluorescence in treated samples clearly indicates the entry of PI into the bacterial cells through ruptured bacterial membrane as a result of curcumin I treatment. Fig. 3B and 3C represent the number of *S*. *aureus* cells showing PI fluorescence on exposure of 25 μM, 50 μM and 100 μM curcumin I and two positive controls HNP-1 and nisin (well known antimicrobial peptides causing bacterial membrane permeabilization [22]) after 2h incubation, as quantified from a flow cytometry experiment repeated on three different days. As shown in Fig. 3B, the PI positive *S*. *aureus* cells constituted only 22.8 ± 2.8% of the untreated sample, whereas 3 μM HNP-1 and 3 μM nisin showed 35.7 ± 0.55% and 57.1 ± 2.97% cells stained with PI, respectively. However, cells exposed to 25 μM, 50 μM and 100 μM curcumin I for 2h showed 96.4 ± 0.92%, 98.6 ± 0.72% and 96.2 ± 4.6% cells having PI, respectively. The increase in PI positive cells in treated samples was found to be statistically significant when compared to control (p value < 0.001).

The entry of PI in *S*. *aureus* cells due to the exposure to curcumin I as shown by flow cytometry was further established by steady state fluorimetry assay. The incubation of *S*. *aureus* with different concentrations of curcumin I for the chosen time points (30 min, 60 min & 120 min) showed a significant increase in PI fluorescence intensity in comparison to the untreated control samples when measured in the spectrofluorimeter. As depicted in Fig. 3C, the PI intensity was less than 35 a.u. for control *S*. *aureus* cells, i.e. without curcumin I exposure. However, on exposure of 25 μM, 50 μM and 100 μM curcumin I for 30 min, the PI fluorescence intensities increased to 51 ± 6.4 a.u., 60 ± 5.9 a.u. and 67 ± 12.0 a.u., respectively. On further increasing the exposure time, there was ∼ 5%–12% increase in PI fluorescence intensity for all the concentrations of curcumin I tested.

### Membrane permeabilization of *E*. *coli* through PI uptake

**Fig 4:**
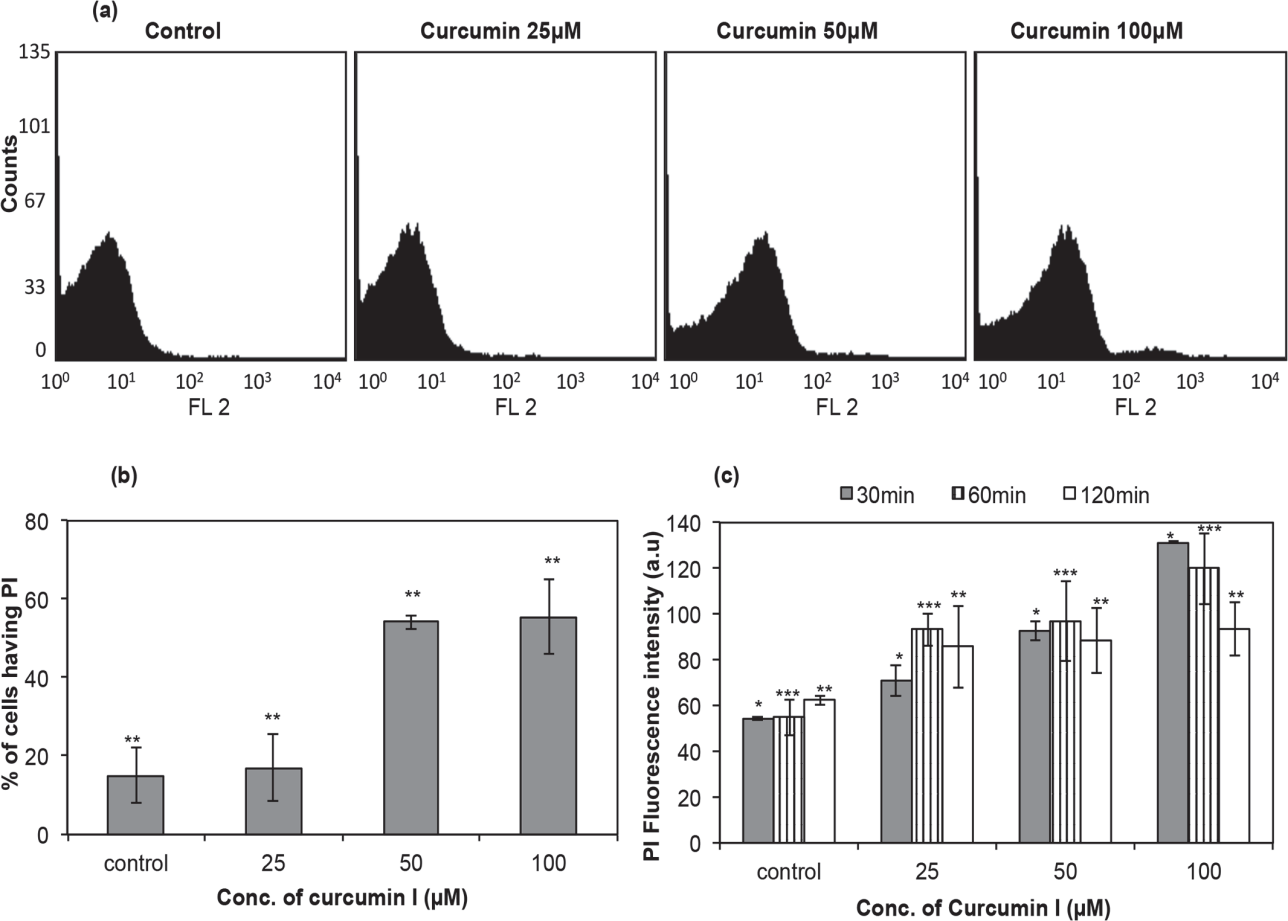
Membrane permeabilization of *E*. *coli* ATCC 25922 by curcumin I, using propidium iodide (PI) through flow cytometry and spectrofluorimetry technique. A total of 10,000 cells were acquired for each flow cytometry analysis. (a) Histograms of cells labeled with PI and treated with different concentrations of curcumin I (0 μM (control), 25 μM, 50 μM & 100 μM) for 2h, (b) percentage of PI uptake by *E*. *coli* cells as quantified from flow cytometry data and (c) fluorescence intensity of PI in *E*. *coli* cells exposed to curcumin I using spectrofluorimetry. These data represent mean (±SD) of three independent experiments (*** p ≤ 0.05, ** p ≤ 0.01, * p < 0.001).

The membrane permeabilization studies using PI were repeated for cells of a Gram-negative bacterial strain, *E*. *coli*. PI uptake in *E*. *coli* cells treated with curcumin I has been represented in Fig. 4A–4C. Fig. 4A shows shift of fluorescence peak towards right (FL2 >10) in histograms of 25 μM, 50 μM and 100 μM curcumin I treated cells compared to untreated cells. The quantitative analysis of three days of flow cytometry data has been presented in Fig. 4B; cells exposed to 25 μM, 50 μM and 100 μM curcumin I for 2h resulted in 17 ± 8.46%, 54 ± 1.74% and 55 ± 9.5% PI staining, respectively, compared to unexposed control cells (15 ± 6.99%).

Spectrofluorimetry also confirmed the membrane leakage of *E*. *coli* cells (Fig. 4C). PI fluorescence intensities of 71 ± 9.7 a.u., 93 ± 6.1 a.u. and 131 ± 0.6 a.u. were observed when *E*. *coli* cells (Fig. 4C) were exposed to 25 μM, 50 μM and 100 μM of curcumin I, respectively, for 30 min, in comparison to control (54 ± 0.7 a.u.). However, after 2h of exposure to curcumin I, the PI intensity decreased. This may be due to complete lysis and leakage of cell content from *E*. *coli* cells.

### Membrane permeabilization by calcein leakage assay using flow cytometry

**Fig 5:**
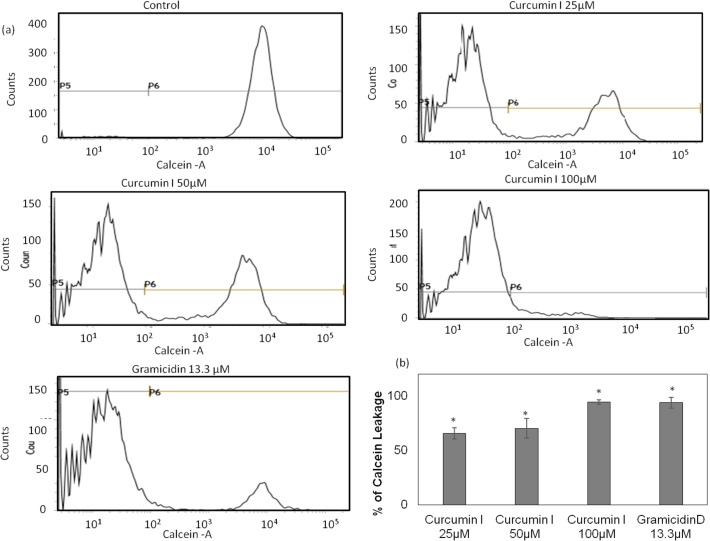
Membrane permeabilization of *S*. *aureus* by curcumin I using calcein-AM through flow cytometry. Logarithmic *S*. *aureus* ATCC 29213 was labeled with calcein and analyzed for membrane permeabilization after incubation with different concentrations of curcumin I. A total of 10,000 cells were acquired for each flow cytometry analysis. (a) Histograms showing calcein leakage, on treatment with different concentration of curcumin I (0 μM (control), 25 μM, 50 μM & 100 μM), (b) percentage of calcein leakage from *S*. *aureus* cells on treatment with different concentration of curcumin I for 2h as quantified from different days of flow cytometry data. These data represents mean (±SD) of three independent experiments (*p ≤ 0.001).

The leakage of membrane by curcumin I was further verified, with the help of a distinct fluorescence probe, calcein and presented in Fig. 5A–5B. Histograms generated from the flow cytometry data (Fig. 5A) indicate calcein-loaded untreated control *S*. *aureus* sample (100% cells showing fluorescence between 10^3^–10^4^ a.u.). In comparison, curcumin I treated samples showed loss in calcein positive cells in a dose dependent manner (fluorescence intensity < 10^2^ a.u.) (Fig. 5A). Gramicidin D was incorporated as a positive control in this assay, which caused loss of calcein from 94% of cells as a result of membrane pore formation [19, 20]. Furthermore, the percentage of calcein leakage was quantified from the flow cytometry data repeated on different days (Fig. 5B). The exposure of *S*. *aureus* cells to 25 μM, 50 μM and 100 μM curcumin I for 2h led to 65.5 ± 5.2%, 70.3 ± 9.15% and 94.4 ± 1.93% calcein leakage, respectively (Fig. 5B). The loss in calcein fluorescence as a result of membrane permeabilization in curcumin I and gramicidin treated samples as compared to the untreated control samples was found to be statistically significant (p value < 0.001).

### Morphological changes in bacteria on curcumin I exposure

**Fig 6:**
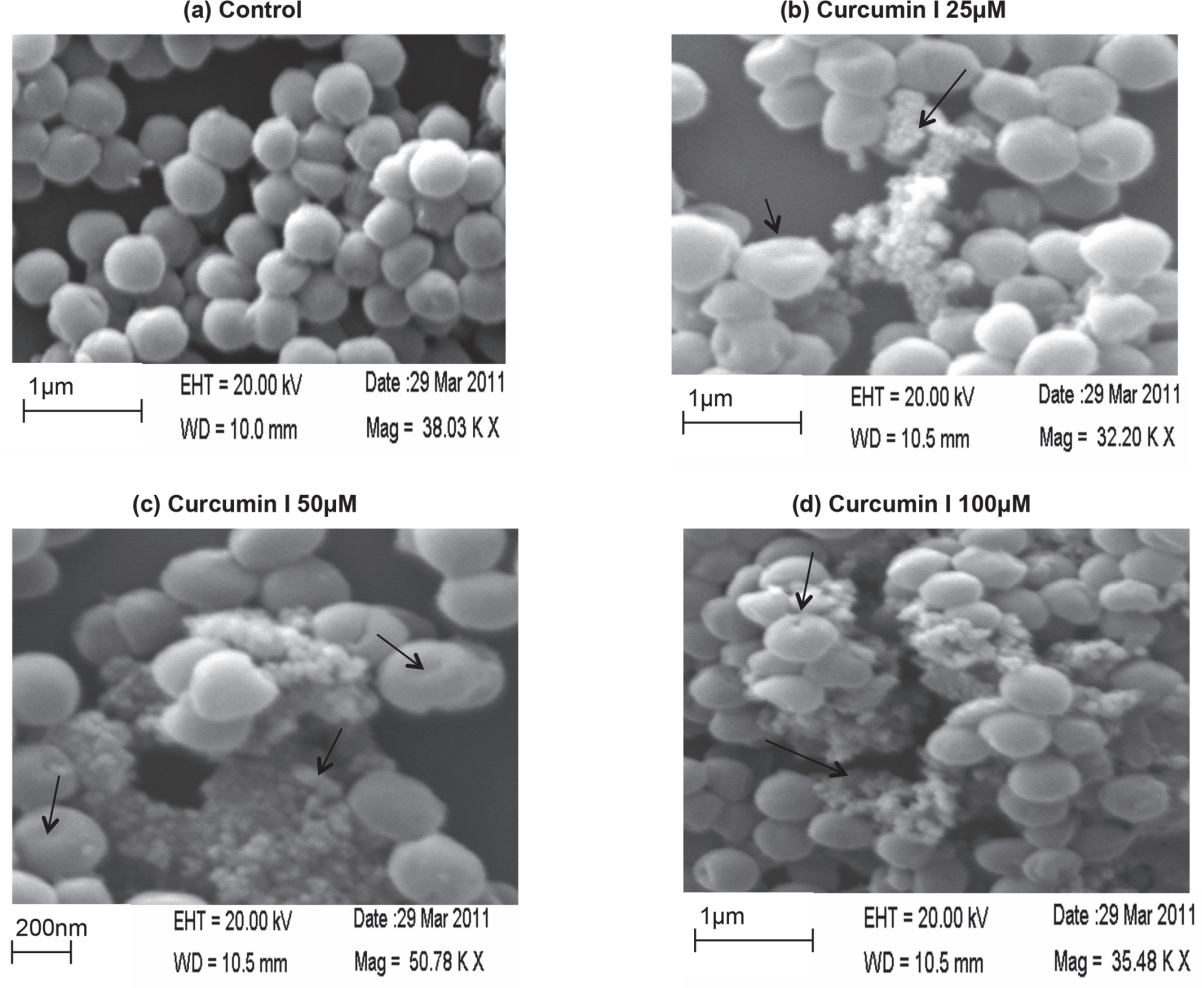
Scanning electron microscopic images of *S*. *aureus* cells after 2h exposure to curcumin I. (a) 0 μM, (b) 25 μM, (c) 50 μM, & (d) 100 μM curcumin I. *S*. *aureus* cells exposed to lower concentration of curcumin I show morphological changes including surface roughness, depression, and dent formation, whereas incubation at the higher dose caused leakage of cell material and bursting of cells which were absent in untreated control cells. Similar appearances were found in separate experiments on different days.

To examine the effect of curcumin I on bacterial morphology, curcumin I treated *S*. *aureus* samples were viewed in SEM and the images of treated cells were compared with the untreated control specimens (Fig. 6A–6D). Fig. 6B–6D represents the images of 25 μM, 50 μM and 100 μM curcumin I treated *S*. *aureus* cells, respectively. As can be seen from Fig. 6B–6D, there is distortion in the shape of cells, with depressions on the surface as a result of exposure to curcumin I. The frequency of dead and damaged cells was found to be more at higher concentrations (i.e. 100 μM of curcumin I exposure). However, the untreated control cells were found intact and devoid of any such alterations (Fig. 6A).

### Membrane damage and death of *S*. *aureus* due to curcumin I as assessed by fluorescence microscopy

**Fig 7:**
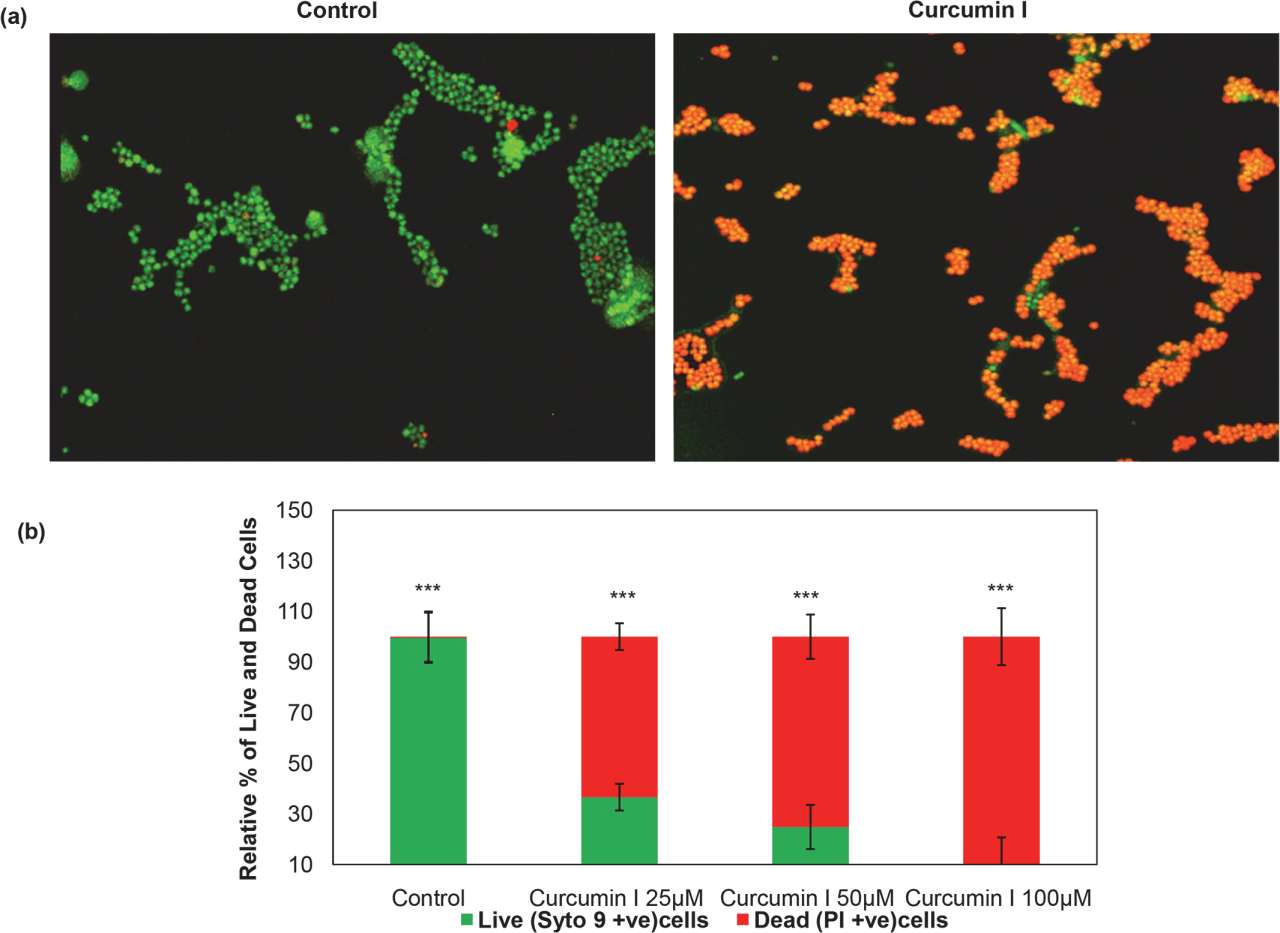
Fluorescence microscopy assay for viability of *S*. *aureus* against curcumin I. Bacteria were exposed to 25 μM, 50 μM & 100 μM curcumin I and saline (negative control) for 2h at 37°C. Subsequently, bacterial sets were washed in saline and subjected to LIVE/DEAD *Bac*Light Bacterial Viability Assay. (a) Microscopic images of representative curcumin I treated and untreated bacterial cells, bacteria in red are indicative of dead or membrane damaged cells, whereas, green indicates live/healthy bacteria, (b) relative percentage of live and dead cells in sample exposed to 25 μM, 50 μM & 100 μM curcumin I and saline (control) as quantified from different microscopic images (*** p ≤ 0.05).

Curcumin I induced membrane damage of cells was visualized by epifluorescence microscopy using fluorescent probes SYTO 9 and PI which selectively stain live and dead cells, respectively. The representative microscopic images (a merged view of both channels) of control (untreated) and curcumin I (25 μM) treated have been presented in Fig. 7A. It can be clearly seen from Fig. 7A that untreated control *S*. *aureus* cells appeared predominantly green (demonstrating live cells); whereas the curcumin I treated cells appeared substantially red (indicating dead cells). The percentage of live (syto 9 stained) and dead (PI stained) cells in all selected doses of curcumin I as well as control was quantified from the microscopic images, using Olympus fluoview version 2.1. Fig. 7B represents the dose dependent increase in PI staining (red fluorescence) on curcumin I (25 μM, 50 μM & 100 μM) exposure. For example, 25 μM, 50 μM and 100 μM curcumin I exposure resulted in 63%, 75% and 90% dead cells, respectively, however, control showed only 0.4% dead cells.

## Discussion

Curcumin I is the most active component of turmeric that has been explored for its various biological and medicinal properties [1, 5, 23]. In the present study, we focused upon the antibacterial activity of curcumin I against four genera of bacteria, including those that are Gram-positive (*S*. *aureus* and *E*. *faecalis)* and Gram-negative (*E*. *coli* and *P*. *aeruginosa*). Being an amphipathic and lipophilic molecule, curcumin inserts into liposome bilayers and enhances their permeability [24]. Besides, it has also been found that curcumin is involved in disordering the 1,2-dipalmitoyl-sn-glycero-3-phosphocholine (DPPC) membranes [25]. All previous studies on curcumin have indicated its membrane altering properties such as thinning and disruption of the membrane at high concentrations using artificial membranes only [26]. However, curcumin induced membrane permeabilization was never studied exclusively using live bacteria from either Gram-positive or Gram-negative groups, which is the main focus of our study. The level of bacterial infection is directly related to the level of inoculum size of the bacterium and infections with high densities of bacteria are resistant to antibiotics [27]. Therefore, in order to critically evaluate the antibacterial efficacy of curcumin I, the killing assay was performed using both low (10^4^ CFU/ml) and high (10^6^ CFU/ml) cell densities of *S*. *aureus* and *E*. *coli*. Moreover, all of the membrane permeabilization assays were performed using 10^6^ CFU/ml, as high cell number is a requirement of the flow cytometry technique. Thus, in the present study, we specifically investigated the effect of curcumin I on bacterial cell membranes at high cell densities and explored the permeabilization of bacterial membranes induced by curcumin I.

The present study found that curcumin I was equally effective against all the tested bacterial genera from both Gram-positive and Gram-negative groups, and the extent of killing showed an increase with dosage and incubation time. We observed a 100% killing at a dosage of 100 μM curcumin even when higher bacterial density (10^6^ CFU/ml) was used. These observations are in agreement with previous studies, which showed an 80% decrease in *E*. *coli* cell growth on exposure of 100 μM curcumin [15], 100% inhibition of *S*. *aureus* and *P*. *aeruginosa* [8], and 80% inhibition in case of *E*. *faecalis* [8] due to the exposure of 200 μM of curcumin within 4h. The high concentrations required to kill the bacteria in this and other studies may be due to poor solubility of curcumin in aqueous media and the low bioavailability of curcumin [28]. To deal with this issue, several bio-conjugates and nanoparticles of curcumin have been synthesized by other groups and are under investigation [28–30].

In the present study, we examined the loss of integrity of the bacterial membrane due to curcumin I exposure, using two different fluorescent probes, PI and calcein. As our results demonstrate, bacterial cells treated with curcumin I have leaky membranes allowing for greater PI entry or higher calcein release. The disruption of the membrane leading to PI uptake in curcumin I treated cells was further supported by both flow cytometry and steady state fluorescence assays. Fluorescence microscopy studies using LIVE/DEAD kit also confirmed the excessive entry of PI molecules in Staphylococcal cells due to membrane damage by curcumin I. Moreover, confocal microscopy images demonstrated PI incorporation into non-viable/dead cells, indicating membrane damage of both Gram-positive and Gram-negative bacteria. SEM images of curcumin I treated specimens clearly showed membrane damaged cells turning to debris. Data of killing and membrane permeabilization assays (PI uptake & calcein leakage) taken together show that a 2h exposure to 100 μM curcumin I causes 100% killing even with 10^6^ CFU/ml bacterial density accompanied by 94%–98% leakage of cell membranes of *S*. *aureus*. This suggests a clear correlation between bacterial killing and its membrane damage due to curcumin I. Thus, all our experiments confirm that damage of the membrane is a key mechanism of curcumin I mediated killing of *S*. *aureus* and *E*. *coli*. It is worthy to note that curcumin I caused membrane permeabilization of both *S*. *aureus* and *E*. *coli* despite their significantly different cell wall properties. No such previous reports exist, which show that curcumin I attacks both Gram-positive and Gram-negative bacteria in a similar manner by causing membrane permeabilization.

The membrane permeabilizing activity of curcumin I, as demonstrated in this study, may be exploited in future to increase the efficacy of obsolete antibiotics by enhancing their cellular uptake. Studies are being conducted in our laboratory to test this hypothesis. Such a strategy could be immensely promising in the current scenario of continuous emergence of resistant microorganisms and antibiotic crisis [31, 32]. Oxidative stress and inflammation often go together with bacterial infections [33, 34]. Therefore, curcumin I, an established anti-oxidant and anti-inflammatory natural herbal product, with easy availability, high efficacy, and low cytotoxicity, could be an ideal candidate to use in combination therapies against bacterial pathogens of humans.
